# A case for environmental statistics of early-life effects

**DOI:** 10.1098/rstb.2018.0110

**Published:** 2019-02-25

**Authors:** Willem E. Frankenhuis, Daniel Nettle, Sasha R. X. Dall

**Affiliations:** 1 Behavioural Science Institute, Radboud University, Nijmegen 6500 HE, The Netherlands; 2 Centre for Behaviour and Evolution and Institute of Neuroscience, Newcastle University, Newcastle upon Tyne NE1 7RU, UK; 3 Centre for Ecology and Conservation, University of Exeter, Penryn TR10 9FE, UK

**Keywords:** evolution, development, environmental statistics, early-life effects, sensitive periods

## Abstract

There is enduring debate over the question of which early-life effects are adaptive and which ones are not. Mathematical modelling shows that early-life effects can be adaptive in environments that have particular statistical properties, such as reliable cues to current conditions and high autocorrelation of environmental states. However, few empirical studies have measured these properties, leading to an impasse. Progress, therefore, depends on research that quantifies cue reliability and autocorrelation of environmental parameters in real environments. These statistics may be different for social and non-social aspects of the environment. In this paper, we summarize evolutionary models of early-life effects. Then, we discuss empirical data on environmental statistics from a range of disciplines. We highlight cases where data on environmental statistics have been used to test competing explanations of early-life effects. We conclude by providing guidelines for new data collection and reflections on future directions.

This article is part of the theme issue ‘Developing differences: early-life effects and evolutionary medicine'.

## 1. Introduction

Early-life effects are widely observed in nature, from tiny *Daphnia* to long-lived humans. Strictly, early-life effects are defined as cases where an input early in life has a larger effect on the adult phenotype than the same input occurring later in life [1]. In practice, the comparison with the same input occurring later in life is rarely made, and so early-life effects simply denote cases where an early input produces a substantial and enduring impact on the adult phenotype. The term ‘early life’ itself refers to the period from conception to the end of juvenile growth and the onset of sexual maturation [2].

Early-life effects are thus phenotypically plastic responses that depend on a sensitive period—i.e. a period in which experience shapes phenotypic development to a larger extent than other periods [1,3]—in the prenatal or juvenile life stage. Early-life effects are not inevitable: some bird species learn new songs throughout their lives and others only in their first weeks [4]. They are not uniform: members of the same species lose their plasticity at different rates [5]. Nor are they general: plasticity trajectories differ between traits within a single individual [6]. What explains variation in early-life effects between species, individuals and traits? Why are early-life effects irreversible in some cases, but not in others?

In recent decades, there has been major progress in our understanding of the neural-cognitive mechanisms of early-life effects [7]. It is now possible to modify aspects of early-life effects, such as their onset, offset and duration, for a variety of traits (e.g. sensory, cognitive and stress response systems) in a variety of species—including non-human primates, rodents and birds—through environmental or pharmacological manipulation. This work holds great promise for future interventions; for instance, by enabling erasure of signatures of trauma. Despite such progress, we know little about the ultimate evolutionary pressures that shape the proximate mechanisms producing early-life effects [1].

### (a) Constraint or adaptation?

A conventional view in both biomedicine and in behavioural ecology is that early-life effects reveal constraints on available resources for development. That is, the early-life input deprives the developing organism of a critical resource (or lifts a resource constraint), resulting in an adult phenotype that is of lower (or higher) quality than it would otherwise be (a ‘silver spoon’ effect; [8,9]). However, constraints and silver spoons cannot explain all early-life effects [10–12]. For example, in zebra finches, early-life exposure to heat stress may increase adult survival, but only when heat stress is encountered again in adulthood [13]. Explanations of such early-life effects are based on two ideas: first, there are conditional adaptations (*if* the environment is hot, then a certain phenotype enhances fitness, but otherwise it does not); and second, early experience carries *information* (if it is hot now, early in ontogeny, it is also likely to be hot at later-life stages). Organisms can exploit the information provided by their early-life experiences to better match their phenotypes to their adult conditions. This process has been likened to a ‘weather forecast’ [14]. How widespread such early-life effects (known as external predictive adaptive responses, PARs) are, which cases are convincing examples, and what exactly it is that the organism is forecasting are much-debated topics [15–19].

One key resource for making progress on the question is theory. There has been a considerable proliferation of formal theory dealing with adaptive early-life effects (e.g. [15,17,20–31]). These models all find conditions under which it could potentially be adaptive to use early experience to set the adult phenotype. However, whether it is fitness-enhancing to do so or not always depends on the assumed statistical properties of the environment, as well as assumptions about the properties of the organisms. Indeed, much of the focus in this theoretical work is on identifying those properties of environments and features of organisms that would make informational early-life effects potentially adaptive.

### (b) Bridging theory and data

To date, the link from theory to empirical evidence has not been strong. That is, although the models suggest that whether or not a particular early-life effect could be adaptive depends on statistical properties of the environment, few empiricists invest in measuring these properties. For instance, Uller *et al.* [32] carried out a meta-analysis of experimental studies of anticipatory parental effects, where the environment experienced by the parent affects the phenotype of the offspring. Formal theory suggests that, if such effects are really informational, they should only be expected where environmental conditions are correlated across generations, so that the experience of the parent provides information about the likely experience of the offspring. Uller *et al.* [32] found that only 7 of the 58 studies they reviewed provided data, or cited papers including data, about whether such correlations actually existed for that species in the wild. As Burgess & Marshall note: ‘in the absence of explicitly estimating the reliability of environmental cues, the adaptive significance of plasticity remains unclear’ [33, p. 2329].

Mathematical modelling can elucidate what processes and outcomes to expect depending on different conditions. However, only empirical data can teach us what conditions actually apply to particular species or taxa. At present, for the vast majority of species, there is a dearth of data on environmental statistics in the wild, or else those data have not been integrated into the study of early-life effects. The aims of this paper are to make a case for greater attention to the statistics of environments and to suggest sources of evidence where they already exist.

There have been prior excellent calls for quantifying environmental statistics. In particular, Burgess & Marshall [34,35] have analysed the role of environmental predictability in shaping adaptive maternal effects and the evolution of life histories, formally and empirically. Because of their focus on maternal effects in particular, their analyses emphasize the statistics of non-social environments across generations, such as correlations between parent and offspring conditions (e.g. in temperature or rainfall). The current paper, by contrast, emphasizes statistics of social environments within generations; in particular, cues to the present conditions and correlations between social conditions experienced early and later in life. We make only one excursion to intergenerational transmission of resources (in §3c). As a consequence, we do not discuss parent–offspring conflict and assignment of fitness to parents and offspring [36]; but rather, we discuss data on the statistics of social environments and the processes that give rise to these statistics. In addition, we draw on examples from human research more than previous work has done. Despite our different starting points, there is some convergence in conclusions with the work of Burgess & Marshall [34,35].

We first discuss recent theoretical models of the evolution of early-life effects (§2). Then, we briefly review empirical research on environmental statistics (§3). Next, we discuss several cases where researchers have already drawn on knowledge about environmental statistics to inform their explanations of plasticity, including early-life effects (§4), and provide guidelines for future research (§5). We end with conclusions and future directions (§6).

## 2. Modelling early-life effects

In the past decade, a set of formal models has emerged that explores the optimal decisions of developmental systems that have access to information coming from multiple sources, such as genes, prenatal effects and postnatal experiences. These models are frequently designed within the framework of statistical decision theory [37] and include Bayesian updating [38–43]. Optimal (i.e. evolutionarily stable) decisions, then, are either derived analytically, computed using dynamic programming methods [39,40,44,45], or approximated using reinforcement learning methods [46,47] or simulations [31].

Formal models of early-life effects do not assume a sensitive period; rather, such a period may emerge in some conditions as the outcome favoured by natural selection. A model generates an early-life effect if the expected fitness of the developmental system is maximized when early cues have a greater impact on phenotypic development than later cues do (in the extreme, later cues do not affect the phenotype at all; i.e. a critical period). For this to be possible, models should include at least two time periods in which the developmental system can access cues, which have the potential to shape phenotypic development. Until recently, however, models of phenotypic plasticity typically assumed a two-stage life history: organisms first sample a cue to the environmental state, and then develop phenotypes based on this cue, either instantaneously or after a (fixed or flexible) time lag. In such models, organisms have no opportunity to sample cues sequentially and gradually adapt to their environments. These models, therefore, cannot produce developmental trajectories in plasticity over time (a precondition for sensitive periods), which may depend on experience. Recent models have allowed for such trajectories by modelling development as a sequential information sampling and decision-making process and by allowing organisms to construct phenotypes incrementally. These models have led to new insights and hypotheses about early-life effects, some of which are obvious, and others not. A full review of these models is beyond the scope of this paper (see [1]). Here, we feature some key themes arising from their results.

### (a) Environmental variation

The first theme is that adaptive evolution of informational early-life effects requires stability of the fitness-relevant environment over developmental time. When environments are completely stable within lifetimes (yet variable across generations, otherwise the environment is constant favouring canalized or ‘genetically fixed’ strategies), it is adaptive to use early-life experience as informative about the adult environment: the organism obtains information from sampling in early life and steeply diminishing returns from continuing to sample once it has some information. Hence, plasticity is predicted to decline sharply with age under such scenarios [1,48].

If the fitness-relevant environment is variable within lifetimes, higher rates of within-generation environmental change (i.e. lower temporal autocorrelation) reduce the payoff for using early-life information to set the adult phenotype [15,20,22,24,26,30,31]. In such environments, more recent cues should often be given greater weight than older ones, favouring learning mechanisms that have the potential to overwrite older environmental estimates, rather than irreversible developmental commitment [20,45,49–51]. Thus, if early-life effects exist for environmental dimensions that change much faster than the timescale of development for a given species, they probably do not reflect ‘weather forecasting’ about the external environment [15,26,31]. Some authors have suggested that informational adaptations based on early-life experience are more likely to occur in short-lived than in long-lived organisms. For early-life effects in long-lived species, if individuals are forecasting anything, they might be forecasting the future capacities of their own soma, which may have been constrained by their poor start (an ‘internal PAR’; [18,26]).

### (b) Cue reliability

A second theme is the reliability of cues about the environment. Cue reliability may appear to be the same thing as within-generation environmental change, but the two are not identical. If the environment is fluctuating unpredictably, current experience is necessarily an unreliable cue of future experience [52]. However, current experience may be a more or less accurate indicator of the present conditions even in a stable environment: sensory detection could be inaccurate, and experiences are often only stochastic reflections of environmental parameters they provide information about (e.g. there may be smoke but no fire). In some cases, the cue experienced is different from the environmental parameter that will determine fitness in adulthood: for example, parental behaviour has been proposed as a cue to the child's future socio-environmental conditions [53,54]; and *in utero* nutrition has been proposed as a cue to future food availability [55]. It is easy to see that these cues will be imperfectly related to the outcomes they are supposed to forecast.

In general, less reliable cues should often be sampled for longer (if not ignored altogether), and given less weight, than more reliable ones, unless unreliable cues are used as a way of creating diversified bet-hedging within a lineage [20,25]. Thus, we may expect longer sensitive periods, and weaker effects of a single brief input, for early-life experiences that are only unreliable cues of a fitness-relevant parameter; and shorter sensitive periods and larger effects for highly reliable cues. In addition, the duration of sensitive periods will often optimally depend not on time, but on the informational state of the organism: an individual receiving a consistent set of cues (e.g. all cues indicate the same level of danger) should shut down plasticity sooner than an individual whose experience is inconsistent [23,27].

### (c) Costs of plasticity

The third theme is that, as long as there is some chance of the environment changing or being unreliably ascertained, for early-life effects to be advantageous, there must be some cost to retaining complete plasticity indefinitely. Otherwise, committing to a phenotype on the basis of early experience is at best neutral, and more often disadvantageous, compared to remaining uncommitted. The costs of retaining full plasticity are implemented in various ways in different models. In some cases, switching between adult phenotypic states is assumed to have a negative effect on survival or fecundity [20,22,56]. In others, a temporary state of maladaptation while switching is assumed [30]. Another approach is to stipulate that specialized adult phenotypes require incremental development, which takes time. Alternatively, earlier integration of different components of a phenotype is assumed to increase their coordination and efficiency [57], providing a benefit to committing to early [23,27]. Without these costs or constraints, an optimal organism would be a Darwinian demon: infinitely plastic throughout its life. Thus, although informational accounts of early-life effects are adaptive accounts, they also contain an element of constraint in their reasoning: early-life cues are given such weight because it is costly or impossible to remain completely plastic through all life stages [58].

In summary, the formal models suggest that in assessing whether an early-life effect is likely to result from adaptive use of information, we need considerable knowledge about the statistical structures of environments. It matters how reliable the putative cue is; and it matters to what extent the present is a good guide to future conditions. These within-generation principles converge with those from analyses of the between-generation environmental statistics that favour the evolution of anticipatory parental effects [34–36,52]. The theoretical work challenges researchers to be more specific about exactly which cues they assume developing organisms to be using, what it is that those cues are indicating, and why conditions in the present carry information about conditions in the future. In particular, the current formal models generate a need to gather empirical data on the statistical structures of different dimensions of environments over the life course, to test claims about adaptive early-life effects for a particular species, cue and environmental parameter. This kind of work has only recently begun in the study of early-life effects. However, there are several other literatures also interested in the statistical structure of environments that we can turn to. The rest of the paper is devoted to empirical work on the statistics of environments over the life course: what has already been done, what needs to be done and how it can be done.

## 3. Research on environmental statistics

The models reviewed in §2 show that such parameters as cue reliability and environmental autocorrelation are essential in shaping early-life effects. We now survey three bodies of research that estimate environmental statistics over evolutionary and developmental timescales: fluctuations in population size, density and composition; intergenerational transmission of resources; and lived individual experiences. Note that these statistics are concerned with the social environment in particular. We argue that the social environment may be particularly relevant to the evolution of early-life effects, because it is likely to have the prerequisite properties of variability over evolutionary time, but considerable stability over developmental time. There are also substantial bodies of work on quantifying spatio-temporal variation in non-social ecological parameters such as temperature and rainfall. This work emphasizes, just as we do, the fundamental role that temporal and spatial scale plays in shaping the course of adaptive evolution [59–62]. As this work has been reviewed in detail elsewhere [34,35], we restrict ourselves to a brief recap of the ways in which environmental statistics are quantified in ecology, before turning to research on social parameters.

### (a) Recap: quantifying environmental statistics

An essential statistic is the autocorrelation parameter in environmental time series. Its mean, variance and stability determine the correlation pattern in a time series. This pattern is described as having different ‘colours’. ‘White’ noise has no temporal autocorrelation: the environmental states at any two points in time are independent of each other [63,64]. When environmental states are positively correlated, noise is described as pink, brown or black, depending on the degree of autocorrelation. In modelling a time series, the parameter *r* captures the relative importance of the value in a time period for determining the value in the next time period. The colour of environmental noise is closely connected with the timescale we consider [65]. The relevant timescale depends on the life cycle of the species [64]. For instance, an environment with moderately positive autocorrelation over months will act as white noise over millions of years [66]. Autocorrelation over months is relevant to the genetic adaptation of short-lived animals (e.g. house flies), because this window includes several generations. For longer-lived animals (e.g. elephants), however, months are a mere blip in developmental time. Even if elephants respond to short-term autocorrelation when foraging, they would be unlikely to use it to irreversibly canalize any aspect of their development.

Analyses of environmental time series have shown that marine habitats tend to show higher positive autocorrelation than terrestrial habitats [67], and coastal habitats tend to fall in between [68]. Climatic variables also tend to show positive autocorrelation, with temperature showing higher positive autocorrelation than precipitation [68]. An inverse power law, 1/*f^β^*, approximates the spectral densities of environmental time series, where *β* = 0 yields white noise, *β* = 1 pink noise, *β* = 2 brown noise and *β* < 0 blue noise [64,68]. In the power law function, 1/*f^β^*, parameter estimates are typically in the range 1 < *β* < 2 for marine habitats, 0.5 < *β* < 1 for terrestrial habitats and *β* ≅ 1 for coastal habitats [69]. This means that on average, marine animals are better able to predict the external conditions they will face in adulthood, based on early-life conditions, than terrestrial animals are. And also, land dwellers are better able to predict temperature later in life than precipitation.

Formal models of early-life effects often assume a first-order autoregressive environmental process, in which the ‘memory’ of the environment extends only to the previous time period (the ‘Markov property’: you need only a single value in order to make a forecast of the future), with no possibility for delayed effects (e.g. rainfall affecting current soil condition that determines future germination rates). This results in exponential decay of predictive value over time, where the correlation time *tau*, which equals 1/ln(1/*r*), is the time it takes the system to ‘forget’ its initial condition (i.e. the initial condition has no better predictive value than a number drawn randomly from a Gaussian random variable). Future models should explore the evolution of developmental systems under more realistic noise structures, including second- and third-order autoregressive environmental processes, which follow the less sharply declining power law distribution that is characteristic of natural time series [69–72].

### (b) Population size, density and composition

Fitness depends not only on abiotic conditions, but also on population parameters, such as population size and density. Statistical analyses of hundreds of species across many taxonomic groups and geographical locations indicate that, like abiotic variables, temporal fluctuations in population abundance show reddened spectra, i.e. positive autocorrelation. However, unlike in abiotic conditions, these fluctuations show little difference between marine and terrestrial species [73,74]. For 92% of species, the spectral exponents were in the range of 0 < *β* < 2, with an overall mean of 1.02 (pink noise). Hence, ‘the spectra of population data seem to be considerably redder (with exponents of 0.8–1.2) than those of environmental variables' [74, p. 1044], which have values closer to 0.5 [75]. Thus, organisms may be better able to predict the future abundance of their population, based on their early-life conditions, than their future abiotic conditions. Nonetheless, for both abiotic and population parameters, positive autocorrelation is low enough to amount to white noise over timescales of years or decades [73,74].

Fluctuations in population abundance also show that larger body size, which is associated with longer generation time [76], predicts redder spectra, i.e. higher positive autocorrelation [73,74]. Larger-bodied species may thus be better able to predict population abundance on an annual scale than smaller-bodied species. However, this predictive advantage may well be offset by the fact that the gap between early life and adulthood will be longer for larger-bodied species. So, larger-bodied species may be unlikely to evolve early-life effects tailored to future ecological conditions (low *β*s) and to future population abundance (higher *β*s, but probably not high enough to offset their longer lifespan). Instead, we consider it more likely that larger-bodied species tailor their development to their internal expected future somatic decline, i.e. an internal PAR [18,26]. Irrespective of body size, the fact that autocorrelation tends to be higher in population variables than environmental variables suggests that researchers should consider population dynamics as selection pressures in the evolution of early-life effects.

### (c) Intergenerational transmission of resources

Sociologists, economists and anthropologists have a long-standing interest in intergenerational mobility, i.e. the extent to which social and material capital (i.e. resources) is correlated across generations. If this correlation is 1, parents' resources perfectly forecast their children's. If it is 0, each generation is born anew. Income data across countries over the past centuries indicate a correlation that ranges between 0.15 and 0.65, suggesting that inheritance explains only 2–40% of the variation from one generation to the next [77]. If this were the whole story, we may expect social and material advantages to be erased within three to five generations. However, wealth may actually be more persistent than two-generation estimates suggest, with some scholars estimating correlations in the range 0.70–0.75 over five generations [77,78]. Wealth is predicted by grandparental wealth, even after controlling for parental wealth. There is controversy over the processes that explain this second-order autocorrelation process [77,78]. We limit ourselves here to the observation that at least in modern societies, wealth is predictable over several generations, despite much noise from one generation to the next. We discuss the stability of wealth within lifetimes in §4.

Does this observation generalize across human cultures? Borgerhoff Mulder *et al.* [79] studied 21 historical and contemporary populations characterized by diverse economic systems: hunter–gatherers, horticulturalists, pastoralists and agriculturalists. They also examined three types of wealth: material, embodied and relational. Their findings show that wealth persistence varies by economic system. Specifically, ‘intergenerational transmission of wealth and wealth inequality are substantial among pastoral and small-scale agricultural societies (on a par with or even exceeding the most unequal modern industrial economies), but are limited among horticultural and foraging peoples (equivalent to the most egalitarian of modern industrial populations)’ [79, p. 682]. These differences may exist because material wealth is more often transmitted in pastoralist and agricultural societies than it is in horticultural and foraging societies. This cross-cultural study used two-generation estimates. Longer-term autocorrelations may be higher than one would expect based on these estimates [77].

In summary, it appears that in all human societies, wealth is heritable to some extent, but this extent is quite variable. Stable multigenerational differences in family status exist in some other primates, such as baboons, as well [80]. Therefore, in some long-lived primates, the persistence of social and material capital may have been stable enough over a few generations to predict individuals' adult experiences based on their childhood social positions, although only to a limited extent. Such predictions may be more likely to have shaped our species’ developmental systems than predictions about ecological and population variables, which appear to be even more unpredictable. Current research, however, focuses on the dynamics of either ecological or social variables in isolation. Future work could explore how the statistics of *non-social* environments might affect the evolution of *social* structure; as is suggested, for instance, by the observation that bird species that inhabit more unpredictable environments (characterized by higher among-year variability in precipitation) are more likely to breed cooperatively, potentially as a strategy to buffer against risk [81].

### (d) Lived individual experiences

Researchers have also quantified environmental statistics over short timescales, capturing a segment of an individual's lifespan in great detail. Biologists, for instance, have used wearable devices (typically, animal-borne cameras) to register the visual experiences of animals in natural habitats [82–84]. They have also used isotopic signatures of tissues, which integrate diet over the period in which these tissues were synthesized, to uncover parameters of animals' diets (and, by extension, of their ecology) over different timescales. For instance, in fur seals, plasma, red blood cells and whiskers integrate diet over the last few days, weeks and years, respectively [85]. The turnover rates of various proteins in tissues thus reveal the spatial and temporal stability of feeding ecologies, which shape the costs and benefits of early-life effects.

Psychologists have equipped infants with wearable devices, such as headcams or language recorders, in order to document their experiences during unconstrained everyday activities (e.g. [86–88]. This work emphasizes that experience is selective (depends on location and focus), state-dependent and variable between individuals. Smith *et al.* [89] distinguish between three spatial scales. The *third-person view* captures the potential environment, i.e. all perceivable aspects of the environment (in ‘viewshed analysis’ in ecology, this is referred to as the potential visual space [90]). The *first-person view* captures the available environment, i.e. the scene in front of an agent's sensory organ, which depends on the agent's current location, size, posture, activity, and so on. *Fixations*, often measured using eye-tracking, capture focus within the available environment [91].

Data at each spatial scale are relevant to formal models of early-life effects. The potential environment determines the extent to which individuals could have different experiences. For instance, more complex environments offer greater scope for variation in experience between individuals. The available environment affects what experiences different individuals are likely to have. For instance, smaller individuals may be less successful at detecting food, or be more frequently challenged by dominant conspecifics, and therefore experience harsher conditions than larger individuals, resulting in developmental differences (e.g. reduced ability to invest in plasticity). Fixations select what information enters the mind for further processing, which influences estimates about the environment, which may shape development.

High-precision data obtained using wearable devices have the potential to be informative about cue reliability as well as environmental autocorrelation. For instance, in harsher environments, parents tend to have less time and fewer resources available to invest in their offspring [92]. From the child's perspective, therefore, parents may be less responsive to their needs, show anger more frequently, and so on. Wearable devices can be used to examine the differential frequencies of these experiences in different environmental conditions (e.g. as a function of objective measures of local morbidity–mortality rates). To do this, data over short timescales are informative, as long as the data are collected across a variety of environmental conditions. To quantify autocorrelation of experiences, however, we need data collected over timescales longer than those typical in current studies using wearable devices. We look forward to future studies that measure the lived experiences of animals over their entire juvenile periods, or even longer, as these will provide a rich source of information relevant to formal models of early-life effects.

**Figure 1.**
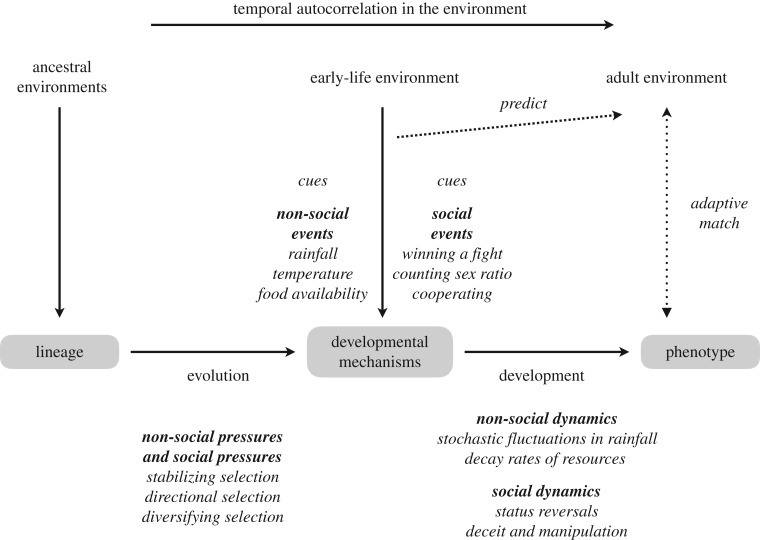
Developmental mechanisms use social and non-social cues to adapt organisms to their current and future conditions. These mechanisms have been shaped, across generations, by selection pressures that depend on temporal autocorrelation in the social and non-social environment. Within generations, developmental mechanisms are also exposed to temporal autocorrelation in the environment, which depends on social and non-social dynamics. Any adaptive evolutionary account of an early-life effect needs to specify each link in the argument and provide evidence that the assumed covariances actually exist in ancestrally relevant environments.

To summarize §3, there are already bodies of literature dealing with the statistical properties of environments. These suggest that some environmental parameters (demographic and social) may show greater stability over a lifetime than others (e.g. rainfall), and moreover that adaptive early-life effects may be more likely to evolve in some kinds of environments (marine) than others (terrestrial). In the case of early-life effects in long-lived organisms, such as humans, claims about whether sufficient temporal stability exists for organisms to use early life as a forecast have proved contentious [15–19]. This section has challenged those who advocate such claims to specify which parameters of the environment they assume organisms to be adapting to, and show that these parameters do have the right kind of temporal stability. Similarly, when researchers make claims that some experience (e.g. parental behaviour) is a cue of some environmental parameter (e.g. harshness), they need to refer to data from which the reliability of such a cueing relationship can be computed. In short, researchers need to specify and justify the assumed linkages in terms of the roadmap for the evolution of adaptive early-life effects shown in figure 1.

In fact, the situation may be even more complex than figure 1 implies. For many social parameters (e.g. relative strength), the social environment may respond dynamically to the phenotype that the focal individual adopts. For instance, an animal who competes successfully over resources may develop a larger body, increasing the probability that conspecifics will defer in future conflicts, with potential associated gains in social status. Or, an animal who successfully manipulates the information used by conspecifics to guide their behaviour may achieve relatively high fitness, increasing the proportion of skilled mind-readers in future generations [93]. The social strategies of animals thus co-determine the statistics of their own social environment, not only because they actively select certain habitats or events (e.g. to enter a conflict or not)—that happens with non-social strategies, too (e.g. a bold forager may explore new terrain)—but rather, because the statistics of social environments respond to the phenotype of the focal individual. This kind of feedback is pervasive in the social world. Although there are, of course, extensive game-theoretic literatures on social dynamics, both within and between generations, exploring winner–loser effects (e.g. [94]), reproductive skew and cueing for mating opportunities (e.g. [95]), honesty and deceit in communication (e.g. [96]), and the coevolution of local relatedness and helping behaviour (e.g. [97]), this type of model does not focus on the statistics of social environments and the processes that generate these statistics. Future modelling should explore this further, drawing on, and informing, empirical research on animal societies; for instance, by drawing on parameter values inferred from studies of the stability of social indices, such as rank or mate value, within and between generations. Such work could support or falsify our speculation, based on the data on population parameters and studies of primates, that social parameters show greater temporal autocorrelation than non-social ones.

## 4. Applications of longitudinal data

There are several cases where researchers have already used environmental statistics to refine their explanations for observed patterns of plasticity, including early-life effects. This is generally only possible in field studies with rich longitudinal datasets. For example, in their study of roe deer, Douhard *et al.* [98] examined the extent to which environmental conditions in an individual's first year of life predicted those in their breeding years. They found that early-life conditions had substantial predictive power in one of their field sites, but essentially none in the other. Thus, to the extent these sites were representative of the environments to which roe deer are adapted, it seems unlikely that the deer would have evolved to use their early experience to calibrate their phenotypic strategies. Indeed, the researchers found no evidence that they did. Unlike Costantini *et al.*'s [13] study of zebra finches, deer exposed to poor early conditions did not fare better if conditions were also poor in adulthood; this ‘match–mismatch’ pattern is often seen as a key prediction of external PAR hypotheses about early-life effects (see below).

In a longitudinal study of Assamese macaques, Berghänel *et al.* [99] studied environmental statistics relevant to both cue reliability and temporal autocorrelation. They showed that maternal stress hormone levels covaried with current environmental conditions; hence, maternal stress hormones are a cue to *current* conditions that the developing fetus could use. However, the researchers also found essentially no temporal autocorrelation in environmental parameters such as rainfall or food abundance. Thus, environmental conditions in early life could not provide information about these aspects of the environment in adulthood. The researchers did find that maternal stress hormone exposure caused accelerated growth at the expense of skill acquisition and immune function, suggesting that the monkeys responded potentially adaptively to early conditions. However, given the lack of temporal autocorrelation, this cannot have been because they were using maternal stress hormones as a cue to future rainfall or food abundance. Thus, either maternal stress hormones provide information about some other, unmeasured environmental parameter, or the developing monkeys were responding adaptively to their own constrained phenotype, i.e. an internal PAR [18,26]; or, there may be no adaptive reason directly related to the environment.

For humans, it has been argued that environmental stability as measured by, for example, climatic or food abundance variables, is unlikely to be sufficient to support the evolution of informational early-life effects [15,19]. However, we suggested in §3 that social parameters might show greater temporal autocorrelation than non-social ones. Nettle & Bateson [100] examined the extent to which socioeconomic conditions in childhood predicted those that will be experienced in adulthood in British women. In line with other findings from affluent societies, they found considerable persistence of socioeconomic position (correlations between the childhood and adult measures of around 0.35). However, they found no evidence of the ‘match–mismatch’ pattern that would be predicted if people could use low childhood socioeconomic position as information and develop an adaptive phenotype to cope with low socioeconomic position in adulthood. Instead, they found that low adult socioeconomic position was even more negative for health if individuals had also experienced low childhood socioeconomic position. This suggests that ‘silver spoon’ effects, whereby good early conditions allow greater overall robustness, dominate over informational adaptation in this instance (see [101,102] for similar patterns in other societies). That said, individuals who grow up in unfavourable circumstances might still be making ‘the best of a bad job’ [9,16], i.e. their fitness outcomes may be better than those of individuals growing up in the same conditions who do not show the same responses as they do. This comparison is challenging to study, especially in wild populations, as it requires somehow blocking the set of responses that animals would normally mobilize in high-adversity contexts.

Just as field datasets can provide evidence on temporal autocorrelation of environmental parameters, they can be used to examine the cues available to developing organisms. Godoy *et al.* [103] used rich observational data from white-faced capuchins to explore the extent to which developing individuals might have access to valid cues of relatedness. They found that the combination of spatial proximity and high status was highly informative about which individuals were their fathers; and spatial proximity and age similarity were strong cues of patrilineal sibship. Thus, early-life adaptations in social or reproductive behaviour contingent on relatedness would be able to make use of these cues. Whether the monkeys do use them was not explored in that particular study. In a different study, however, Godoy *et al.* [104] were able to demonstrate that capuchin monkeys avoid mating with close kin, both at the parent–offspring and half sibling level, and moreover, provided evidence of fitness costs to inbreeding in those cases where it did occur (i.e. delayed age of first reproduction). Furthermore, in humans, it is known that individuals use early-life association with the same female caregiver as a cue of relatedness, probably for purposes of inbreeding avoidance in adulthood [105].

To summarize §4, field researchers have begun to assemble and report environmental statistics relevant to testing accounts of early-life effects. These datasets shed light both on issues of cue reliability and availability, and temporal stability. We argue that more data of this kind are required, comparing across different environmental parameters, different environments and different species. Ideally, multiple replicate datasets are needed, which can be compared and integrated. For an external PAR to evolve, an informational relationship needs to exist not just fleetingly or at some sites, but enduringly, on average, over evolutionary timescales [58].

## 5. Guidelines for new data collection

In a recent survey of the current state of understanding of spatial and temporal variation in ecology, notable gaps in knowledge were highlighted [60]. It is particularly eye-catching that there are substantial gaps in observational datasets at the finest scales of variation (daily to sub-daily timescales and greater than 1 m^2^ to 100 ha spatial scales) that are the most relevant to individual organisms. Nevertheless, given recent technological advances (in wearable tech, remote sensing capabilities, etc.), there are significant opportunities to access the real-world experiences of individuals (both humans and non-human) as they go about their daily lives. Such access should not just be limited to the visual domain as environmental inputs to key developmental processes and systems come in a variety of forms, encompassing all of the ways that a developing organism can be influenced by its environment. Moreover, many non-human animals prioritize non-visual sensory modalities (e.g. most mammals rely on chemosensing more than they do on vision).

For the reasons outlined above, the ecological and evolutionary relevance of the timescales over which statistical variation is quantified must be considered carefully. Key to this will be the generation time of the focal organism. But it will also be important to consider the spatial scale or coarseness of the patchiness in key features of the socio-ecology of the organisms under consideration. For instance, exactly the same environment can be perceived as more or less variable, and any variation more or less stochastic (unpredictable), by organisms of different sizes. Indeed, variation in resource use patterns driven by perceptual scale differences can facilitate the coexistence of species in different size classes on very narrow niches (e.g. single resource types; [106]). Therefore, it will be important to design sampling protocols to the species in question. Furthermore, this issue will limit the value of many of the existing datasets discussed above as they have been collected to be as generally representative as possible, or for other purposes. Finally, the data demands of a full-scale attempt to document the relevant environmental statistics for even a few model species will not be trivial. There will be an increasing need for repositories for open sharing of sensor (e.g. video and sound) files and associated metadata [107]. Moreover, there are likely to be limitations to existing statistical techniques to be overcome, particularly from a spatial perspective. Spatial statistical techniques are notoriously more challenging than their equivalent non-spatial counterparts because spatial data are often subject to severe statistical constraints (e.g. fundamental scale dependency and pervasive autocorrelation; [108]).

## 6. Conclusion and future directions

We hope our paper will strengthen the bridge between formal modelling of early-life effects and empirical research on environmental statistics. We have invited theoreticians to be more explicit about how environmental statistics should be measured to evaluate competing explanations of early-life effects, and to consider building more realistic noise structures into their formal models. Conversely, we have invited empiricists to quantify the environmental statistics suggested by formal models to be important in shaping early-life effects, such as cue reliability and temporal autocorrelation in non-social and social environments, both within and between generations.

Building formal models that incorporate realistic noise structures will be challenging, and even more so would it be to collect, process and analyse rich longitudinal data extending over years or even decades. However, this is feasible if researchers are able to draw on innovative and efficient technologies (e.g. smaller wearable devices with greater storage space and experience-sampling tools). Crucially, rich longitudinal datasets can be used not solely for the purpose of studying early-life effects, but rather for a wide variety of purposes. For instance, video recordings of the visual inputs available to infants provide not only information about the distributions of objects and faces they perceive (the main focus of these studies), but also about the level of contingency of caregiver's responses to their infants. If such recordings are made repeatedly over the juvenile life stage, and at least once in adulthood, we can estimate social environmental statistics, such as the central tendency over time (i.e. slope), variance and stability in caregiving sensitivity, and use these statistics to evaluate competing explanations of early-life effects, including individual differences therein.

So far, we have assumed that environmental dimensions have isolated effects on traits, when, of course, they might interact (e.g. optimal adaptation to temperature may depend on the level of rainfall). Formal evolutionary modelling shows that if a trait depends on multiple dimensions of the environment [109], or on multiple maternal characters [24], optimal reaction norms may differ from their univariate equivalents. For instance, if one maternal trait endures a more predictable form of fluctuating selection than another, this character is likely to disproportionally affect other offspring characters that are adapting to less predictable (noisier) selection, because it provides more information about future conditions [24]. These models, therefore, suggest a need for datasets that simultaneously represent multivariate environments and multivariate phenotypes over time. For the study of early-life effects, within generations, datasets should include multiple measurements over ontogeny and at least one measurement in adulthood. As we noted in §4, certain longitudinal datasets already include this information. We are eager to see such datasets used to parameterize, and test, formal theory.

As in other areas of biology, there is scope for better integration of function and mechanism in the study of early-life effects [110–111]. We have focused on formal modelling and environmental statistics. We have ignored how organisms actually process information and use it to generate actions. As noted in §2, formal models of early-life effects often include Bayesian updating [38–43]. Although this feature does not require that organisms are processing information in a Bayesian manner, it does imply that organisms keep track of environmental cues and respond to them as a Bayesian animal would. This assumption can be questioned on several grounds; here we focus on one. Higginson *et al.* recently showed that ‘animals can achieve a similar level of performance to Bayesians using much simpler mechanisms based on their physiological state’ [112, p. 1], such as energy reserves, which are correlated with fitness-relevant statistics of the environment. Keeping track of the environment takes time and effort, and is presumably costlier than using internal states as a source of information about environmental conditions. When simple ‘rules of thumb’, or heuristics, can achieve high levels of performance, they might well be favoured over strategies that require extensive sampling of multivariate environments. We look forward to future modelling that explores what simple rules of thumb could be favoured in realistically complex environments and thus makes predictions about which kinds of environmental manipulations will produce large plastic responses and which will not.

Finally, it could be helpful if theoreticians and empiricists use similar labels, metrics and graphical representations to describe, quantify and depict environmental statistics. Right now, for instance, empirical articles often show the correlation between trait values and environmental variables at different time points, without reporting the autocorrelation coefficients of the environmental variables themselves, which could be imported into formal models. Consistency will make comparing and integrating among formal models and datasets easier and therefore more likely to occur. We may imagine an ‘encyclopedia of environmental statistics' that details distributions of environmental autocorrelation, and cue reliabilities, over different timescales, documented across different species and within species across habitats, all presented in a standardized format; ideally, accompanied by the raw data. Such an encyclopedia would build on scholarly articles on environmental statistics, but it would have a broader focus; it would integrate these articles into a larger whole. Such a unified overview would offer a scaffold for new insights into the evolutionary pressures and physiological mechanisms that produce early-life effects, setting priors for species or habitats yet to be measured. This encyclopedia would be a valuable resource, continuously updated, helping researchers to discover patterns in a currently mysterious smorgasbord of variation in early-life effects between different species, between individuals within populations, and between different developmental systems within individuals.

## Data accessibility

This article has no additional data.
